# Determinants of Health Promotion Behaviors among Family Caregivers of Stroke Survivors

**DOI:** 10.3390/diseases9010010

**Published:** 2021-01-22

**Authors:** Anna Kavga, Ourania Govina, Petros Galanis, Ioannis Kalemikerakis, Eugenia Vlachou, Nikolaos Fotos, Styliani Tziaferi, Athina Kalokairinou

**Affiliations:** 1 Department of Nursing, University of West Attica, 12243 Athens, Greece; ugovina@uniwa.gr (O.G.); ikalemik@uniwa.gr (I.K.); evlachou@uniwa.gr (E.V.); 2 Nursing Department, National and Kapodistrian University, 11527 Athens, Greece; pegalan@nurs.uoa.gr (P.G.); nikfotos@nurs.uoa.gr (N.F.); athkal@nurs.uoa.gr (A.K.); 3 Nursing Department, University of Peloponnese, 22100 Tripoli, Greece; stziafer@uop.gr

**Keywords:** stroke, family caregivers, health promotion behaviors

## Abstract

Purpose:To record the health promotion behaviors of family caregivers of stroke survivors, as well as potential determinants that could affect these behaviors. Methods: A cross-sectional study was carried out through home visits in the Attica region using the convenience sampling method. The studied population included 109 survivors who had suffered a stroke and experienced functional problems, and their 109 primary caregivers, who were family members, lived in the same house and were fully responsible for their care. The dependent variables were the caregivers’ health promotion behaviors, while the independent variables were the survivors and caregivers’ demographic characteristics, survivors’ functional capacity, depression, social support and changes in caregivers’ lives from caring. Results: Better health promotional behaviors were associated with the following: patient having advanced age and a high level of functionality, caregivers assessing their own state of health as “good”, greater social support, a higher educational level and a higher income level. In addition, more hours of patient care were associated with a less healthy lifestyle for caregivers. Conclusions: Promoting the health of family caregivers of stroke survivors is crucial for both survivors and caregivers. For this reason, it is of great importance to detect factors that affect the health promotion behaviors of caregivers in order to carry out appropriate interventions and improve their quality of life.

## 1. Introduction

Cerebrovascular stroke is one of the world’s leading causes of mortality and disability, for which the financial costs of treatment and out-of-hospital care are particularly high. Indicatively, approximately 14 million new cases of stroke occur annually and 5.5 million patients die from stroke, with 39% of deaths occurring in survivors <70 years of age and 4% in survivors ≤45 years of age [1,2]. In addition, 60% of strokes occur in people aged <70 years and 8% in those aged ≤45 years, while the lifelong risk of stroke among individuals >25 years of age is 25% [2]. 

Moreover, the disability caused by stroke is extremely high, with disability-adjusted life years reaching around 120 million per year [1]. Of all stroke survivors, 40–60% experience moderate to severe disability problems requiring rehabilitation services and/or long-term health care, either institutional or at home [3]. Stroke survivors rely primarily on their families for long-term home health care [4,5,6], but these caregivers may not be prepared to satisfy their patient’s needs. As a result, they face problems such as increased stress and poorer quality of life, depending on various factors, which may include the caregiver’s sex, age and income, among others [7,8,9,10,11,12]. The need for caregivers to be trained in basic care procedures at home is an urgent one, since research has indicated that caregivers who participate in intervention programs can improve their activities of daily living (ADLs) and reduce complications in those being cared for [13].

Health promotion behaviors are an important component of an individual’s quality of life and family caregivers may find these behaviors are negatively affected by their role. Behaviors that promote caregivers’ health and can be negatively affected by the caring role are a healthy diet, physical exercise, stress management, spiritual growth and involvement in enjoyable activities, interpersonal relationships, preserving time and opportunities for health maintenance, undergoing preventive examinations and avoiding tobacco and alcohol use [14].

In addition, caregivers’ health promotion behaviors may be affected by their own self-efficacy, as well as various characteristics of their loved ones, such as their functional capacity, the severity of their condition, social support and family conflicts [10,15,16,17,18]. The severity of a stroke survivor’s condition may lead to depression and high levels of burden, which are common problems for caregivers and lead to a deterioration in their health and their satisfaction with self-care [19]. The factors that negatively affect caregivers’ health, according to Bouer and Souza-Poza (2015) [20], include employment (poor health appears to be connected with both unemployment and overwork), advanced age due to population aging and because of fewer children per couple and the needs that arise from aging itself and from the demands of care [21].

Although few related studies exist, it appears that caregivers of stroke survivors run a greater risk of deterioration in their physical and mental health, manifesting higher rates of stress and depression compared to caregivers of patients with other neurological diseases [22]. The changes following stroke can be severe, and caregivers must adapt to these and adopt a new way of life. The sudden onset of stroke makes it difficult for them to prepare for the caring role. As a result, caregivers may neglect their own personal health and suffer a deterioration in quality of life [23].

For all the above reasons, we conducted a study to evaluate the health promotion behaviors of family caregivers of stroke survivors, as well as possible factors affecting these behaviors.

## 2. Methods

### 2.1. Study Design

This was a cross-sectional study that was carried out through home visits in the region of Attica, Greece. The study population included 109 stroke survivors who had functional problems and their 109 primary caregivers, who were family members and had undertaken their care. The questionnaires were completed by the researchers during home visits to ensure that the questions were understood. Stroke survivors and caregivers were evaluated at home, but in various rooms of the house. The participation rate in the study was 78%, with the main reasons for refusal being a lack of time and the caregiver’s reluctance to participate in the study.

The patient selection criteria were: (a) incapacity for basic or complex daily living activities due to stroke, (b) at least 4 months elapsed since the stroke and (c) the patient should reside at home. The criteria for selecting caregivers were: (a) to be a family member, (b) to have primary responsibility for care and (c) to live in the same home as the patient. The study sample excluded survivors and caregivers who did not speak the Greek language well or had mental or psychiatric problems. The stroke survivors were not directly involved in this study, but served only as part of the recruitment approach in order to identify caregivers, on the basis of the criteria specified above. Stroke survivors’ clinical characteristics and functional capacity were recorded as factors affecting carers’ perceived care burden.

Convenience sampling was performed and the sample was selected from the community in collaboration with neurologists and physicians in the private sector, the home care service of the Hellenic Red Cross, the “NOSILIA” organization and the National Rehabilitation Center. Data collection took place over a period of 13 months. Initially, communication was established and information was provided to the private doctors, the Red Cross nurses and the nurses of “NOSILIA” about the purpose of the research. A positive response was followed by a telephone conversation with the researchers and a visit to the stroke survivor’s home was scheduled. Alternatively, in the case of pairs of stroke survivors and caregivers who were being monitored by the National Rehabilitation Center, the home appointment was arranged by the principal investigator during a visit to the center. During the visit, the patient and the caregiver were verbally informed about the purpose of the study, confidentiality, anonymity, voluntary participation and the possibility of leaving the study at any time they decided; this was followed by the signing of the consent form. Information about stroke survivors’ demographic and clinical characteristics were provided mainly by the caregivers and used by the principal investigator to complete the Barthel Index scale. However, stroke survivors whose general condition permitted it signed the consent form and provided the personal data used in the study. If this was not possible, consent was given only by caregivers who were included in the study by definition and were also the stroke survivors’ legal representatives. The duration of the home visit was about one hour and the time needed to complete the questionnaires was 45 min.

### 2.2. Questionnaires

The dependent variables of the study were the health-promoting behaviors of family caregivers, while the independent variables were the demographic characteristics of survivors and caregivers, survivors’ functional capacity, depression, social support and changes in caregivers’ lives as a result of care provision.

Appropriate questionnaires were used to measure the independent and dependent variables. More specifically, the Health-Promoting Lifestyle Profile II (HPLP-II) scale [24] was used to measure the health-promoting behaviors of family caregivers, and the Barthel Index (BI) scale [18] was used to assess survivors’ functional capacity. The Bakas Caregiving Outcomes Scale (BCOS) [25], which has been validated in the Greek language [26], was used to measure the changes in caregivers’ lives due to the provision of care. The Center for Epidemiological Studies—Depression (CES-D) scale was used to assess depression [27] and the Personal Resource Questionnaire (PRQ 2000) [28] was used to measure social support. The HPLP-II scale includes the subscales of responsibility for health, physical activity, nutrition, mental development, interpersonal relationships and stress management. A higher score on the HPLP-II scale indicates better health-promoting behaviors. The Barthel Index was used to evaluate stroke survivors’ demographic and clinical characteristics as well as their functional capacity [29]. Cronbach’s alpha internal consistency coefficient was >0.7 for all questionnaires, indicating that their reliability was acceptable.

### 2.3. Ethical Issues

Permission was obtained from the Professional Ethics Committee of the Department of Nursing of the National and Kapodistrian University of Athens, Greece to conduct the study in the community. The questionnaires were used after written permission had been obtained from the authors for their translation into Greek and their use in the present study. For access to stroke survivors and caregivers, permission was obtained from the administration of the Hellenic Red Cross (Nursing Department), and from the governing bodies of the “NOSILIA” organization and the National Rehabilitation Center. A necessary condition for participation in the study was that the caregiver should provide signed informed consent; the patient’s consent was also obtained when feasible. Participants were provided with oral and written information about the aims and procedures of the investigation, and the confidentiality of personal information. They were told that their participation was voluntary and that they were free to leave the study whenever they wished. Confidentiality was achieved by not involving any other person in the whole process; the home visit, the completion of the questionnaires, the coding and the recording were all carried out by the researchers.

### 2.4. Statistical Analysis

Quantitative variables were expressed as mean values and standard deviations, while qualitative variables were represented by absolute (N) and relative (%) frequencies. A Student’s *t*-test was used to compare quantitative variables between two groups. Parametric analysis of variance (ANOVA) was used to compare quantitative variables between more than two groups. The Pearson or Spearman correlation coefficient (r) was used to evaluate the relationship between two quantitative variables. Linear regression analysis with a stepwise insertion/removal process was used to find independent factors related to the various scales from which the beta coefficients and their standard errors were derived. Significance levels were two-sided and the level of statistical significance was set at 0.05. The statistical software SPSS V. 19.0 was used for the analysis.

## 3. Results

### 3.1. Demographic and Clinical Characteristics

The survivors’ mean age was 69.3 years; 51.4% were men, 66.1% were married, 89% had children, 35.8% had equipment and facilities at home and 38.5% had an annual family income between EUR 5,000 and EUR 10,000. As regards diagnoses, 52.3% had left and 47.7% right hemiparesis.

The majority of caregivers were women (67.9%), survivors’ spouses (50.5%), married (76.1%), retired (53.2%) and had children (78%). The mean overall duration of care for survivors was 21.8 months and the mean duration of day care was 13.2 h. Caregivers’ rating of their own health judged it to be moderate in 49.5%, good in 40.4% and bad in 10.1% of cases. The characteristics of survivors and caregivers are presented in Table 1.

### 3.2. Health Promotion Behaviors of Caregivers

The Cronbach’s alpha internal consistency coefficient for the HPLP-II questionnaire subscales ranged from 0.72 to 0.91, indicating that the questionnaire had excellent reliability. The descriptive results of the subscales of the HPLP-II questionnaire are presented in Table 2. The total score ranged from 1.69 to 3.17 points, with a mean value of 2.25 ± 0.35. The highest mean scores were for the subscales “interpersonal relationships” (2.66) and “intellectual development” (2.61), followed by the subscales “eating habits” (2.25) “health responsibility” (2.24), “stress management” (2.04) and “physical activity” (1.60).

### 3.3. Correlations

The bivariate correlations between the independent variables and the subscales of the HPLP-II questionnaire are presented in Table 3, while the multivariate linear regressions with the dependent variables of the HPLP-II questionnaire subscales are presented in Table 4.

Due to the big number of variables involved in this study, we chose to list only the statistically significant results. More specifically, caregivers with secondary or tertiary education had a healthier lifestyle overall, compared to caregivers without education or with only primary education (*p* = 0.048), as did those receiving greater social support (*p* = 0.001). In contrast, more hours of patient care were associated with a less healthy caregiver lifestyle (*p* = 0.025). Regarding individual health promotion behaviors, a higher level of social support was associated with greater health responsibility (*p* = 0.013), better physical activity (*p* = 0.003), better nutrition (*p* = 0.005), greater mental development (*p* < 0.001), better interpersonal relationships (*p* < 0.001) and more effective stress management (*p* = 0.008). A higher level of patient functionality was associated with greater health responsibility (*p* = 0.005), better nutrition (*p* = 0.008) and better interpersonal relationships (*p* < 0.001). In addition, more hours of patient care were associated with worse stress management (*p* = 0.007), while caregivers’ better self-perception of health was associated with greater mental development (*p* = 0.045). Compared to uneducated or primary educated caregivers, those with secondary or tertiary education had better eating habits (*p* = 0.016) and better physical activity (*p* = 0.003). Higher income was associated with better nutrition (*p* = 0.002) and survivors’ greater age with better stress management (*p* = 0.003). Male caregivers reported better scores in relation to stress management than female caregivers (*p* = 0.012).

## 4. Discussion

In the present study, the health promotion behaviors of family caregivers of stroke survivors were investigated and various factors were found to influence these behaviors.

The health-promoting behaviors of family caregivers, as evaluated by the HPLP-II scale, were worse than those in the existing literature [30,31,32,33], which indicates that Greek caregivers downplay the importance of their personal health when taking on intensive care roles. This finding may be due to the different populations studied, as well as the fact that health behaviors are not static, but are influenced by various factors, such as social norms, culture, health policies, natural and social environment, etc. The effect of different cultural environments is shown by the variations in health promotion behaviors when the scale has been adapted to languages other than the original English [24,34,35,36].

The present study revealed that a higher level of patient functionality was associated with better health promotion behaviors by family caregivers. Survivors’ functional status, which is not static and changes over time, and the changing needs of caregivers at different stages of the disease, can be a predictor of caregivers’ health behaviors and lifestyles [37]. Factors such as the limitation of activities due to care and changes in personal plans and family life, with changes in the roles they play [38], cause caregivers to experience stress, which increases over time and results in the abandonment of healthy behaviors and a decrease in the quality of life [39]. In addition, stroke survivors’ actual level of health is not always the same as the state perceived by the care provider, i.e., the subjective burden [40], while the relationship between stroke survivors’ level of health and the health of caregivers has not been fully documented [16,40,41].

The present study also found that greater social support was associated with better health promotion behaviors of family caregivers, in all areas of promotion. This finding is also supported by the literature [15,32,42]. Social support is the strongest positive predictor of health promotion and has a positive effect on caregivers’ quality of life [43]. In Greece, social support is greater than in some other countries [44], because of the strong ties that exist between members even of the extended family [45]. It is generally accepted worldwide that the family consists of a group of individuals who are connected to each other by powerful emotional bonds and their own unique cultural perceptions that influence their health [46]. Social support empowers caregivers, helps them remain calm and assists them with the difficult task they are performing, while also acting as a catalyst in providing appropriate health care.

The results of different studies are contradictory as regards differences in health promotion behaviors between caregivers and non-caregivers [47,48]. In the present study, higher educational level and income were found to be associated with better health-promoting behaviors of family caregivers, findings supported by the literature [15,49]. Higher educational attainment is associated with the adoption of a healthy lifestyle and with health-promoting behaviors that improve the caregiver’s biopsychosocial health, while low education is associated with high-risk behaviors such as smoking, obesity, hypertension, high cholesterol, etc. [50]. In the study by Nocon et al. [51], high income and high professional position could not compensate for a low level of education, which shows that it is difficult to change habits after reaching adulthood.

In the present study, as in others [52,53], more hours of patient care were associated with less healthy lifestyles, worse stress management and a poor quality of life for caregivers. This may be explained by the fact that the many hours that caregivers spend caring for stroke survivors limit their time spent on health-promoting activities such as exercise, socializing and maintaining a balanced diet.

The limitations of the study include the convenience sampling, which does not allow the conclusions to be generalized, and its cross-sectional design, which means we cannot draw firm conclusions about the relationship between determinants and health-promoting behaviors, especially in the long term. A future cohort study could investigate the course of health behaviors over time and provide more information about the impact of the burden on caregivers. Finally, the questionnaires used to collect the data carry the risk of all subjective assessments, which may lead to systematic information error.

To conclude, in this study, the factors for caregivers that were found to have a positive influence on health promotion behavior were educational level, social support, self-perception regarding health, income and male sex. As regards the factors related to stroke survivors, a greater functional capacity and older age were associated with caregivers taking greater responsibility for their health and showing better stress management. Promoting the health of family caregivers of stroke survivors is crucial for both the caregivers and the stroke survivors they care for. For this reason, investigating the factors that influence caregivers’ health-promoting behaviors is crucial, so that appropriate interventions can be applied and caregivers’ quality of life can be improved. The implementation of training, information and support programs for caregivers at the primary health care level could play a key role in this direction, helping caregivers to improve their health status and perform their role better. Especially in Greece, future studies of health professionals should focus on the effectiveness of individual interventions using remote means, as these have a low cost, allow the simultaneous empowerment and education of a large number of caregivers and may be continued for a long period. In addition, the value of group interventions should be investigated, since in this way it is possible to empower caregivers to adopt health promotion behaviors and to reinforce their sense of social support.

## Figures and Tables

**Table 1:** Demographic characteristics of the patient and caregiver sample (Ν = 109).

Stroke Survivors N (%)			Caregivers N (%)
Sex	Men	56 (51.4)	35 (32.1)
	Women	53 (48.6)	74 (67.9)
Age	Mean ± SD	69.3 ± 13.7	58.0 ± 13.5
Educational attainment	Uneducated	10 (9.2)	3 (2.8)
	Primary	55 (50.5)	35 (32.1)
	High	31 (28.4)	43 (39.4)
	University	13 (11.9)	28 (25.7)
Family status	Married	72 (66.1)	83 (76.2)
	Unmarried/Divorced/Widowed	37 (33.9)	26 (23.8)
Number of children	Mean ± SD	2.2 ± 1.3	1.7 ± 1.1
Minor Children		7 (7.2)	8 (9.4)
Family members living in the same house Mean ± SD 2.8 ± 1.1			
Family income (EUR)	<5000	20 (18.3)	16 (14.7)
	5000–10,000	42 (38.5)	43 (39.4)
	10,000–20,000	34 (31.2)	36 (33.0)
	>20,000	13 (11.9)	14 (12.8)
Equipment for disabled	Yes	39 (35.8)	
	No	70 (64.2)	
Diagnosis	Right-sided hemiparesis	52 (47.7)	
	Left-sided hemiparesis	57 (52.3)	
Relationship with patient	Spouse		55 (50.5)
	Child		38 (34.9)
	Sibling		7 (6.4)
	Other		9 (8.3)
Employment	Working		33 (30.3)
	Retired		58 (53.2)
	Other		18 (16.5)
Self-perception of health	Good		44 (40.4)
	Moderate		54 (49.5)
	Bad		11 (10.1)
Duration of care (months) 23.7 (26.2) * 10 (5–36) **			
Daily caring hours 13.2 (6.4) *			

* mean ± SD, ** median (interquartile range).

**Table 2:** Descriptive statistics of Health-Promoting Lifestyle Profile II (HPLP-II) subscales.

	Min Value	Max Value	Mean	SD
HPLP-II	1.69	3.17	2.25	0.35
Health Responsibility	1.00	3.67	2.24	0.57
Physical Activity	1.00	3.25	1.60	0.58
Nutrition	1.11	3.44	2.25	0.44
Spiritual Growth	1.67	3.89	2.61	0.43
Interpersonal Relations	1.89	3.89	2.66	0.44
Stress Management	1.38	3.50	2.04	0.40

**Table 3:** Univariate analysis of HPLP-II subscales.

	Health-Promoting Lifestyle Profile (HPLP-II)			Health Responsibility			Physical Activity			Nutrition		
**Stroke Survivors**	**Mean**	**SD**	***p*-Value**	**Mean**	**SD**	***p*-Value**	**Mean**	**SD**	***p*-Value**	**Mean**	**SD**	***p*-Value**
Sex			0.730 ^a^			0.029 ^a^			0.067 ^a^			0.541 ^a^
Male	2.26	0.34		2.36	0.59		1.50	0.49		2.28	0.46	
Female	2.24	0.36		2.12	0.52		1.71	0.65		2.23	0.41	
Educational attainment			0.422 ^a^			0.882 ^a^			0.264 ^a^			0.045 ^a^
Uneducated/Primary	2.23	0.32		2.24	0.52		1.55	0.56		2.19	0.39	
Higher/University	2.28	0.38		2.25	0.64		1.68	0.61		2.36	0.48	
Married			0.336 ^a^			0.777 ^a^			0.399 ^a^			0.325 ^a^
No	2.30	0.34		2.26	0.43		1.67	0.57		2.31	0.43	
Yes	2.23	0.35		2.23	0.63		1.57	0.59		2.23	0.44	
Family income (EUR)			0.164 ^a^			0.959 ^a^			0.277 ^a^			0.001 ^a^
<10,000	2.21	0.35		2.24	0.56		1.55	0.54		2.13	0.42	
≥10,000	2.31	0.35		2.25	0.58		1.67	0.63		2.41	0.41	
Equipment for disabled			0.479 ^a^			0.761 ^a^			0.543 ^a^			0.628 ^a^
Yes	2.22	0.34		2.26	0.49		1.56	0.52		2.28	0.48	
No	2.27	0.36		2.23	0.61		1.63	0.61		2.24	0.41	
Diagnosis			0.342 ^b^			0.392 ^b^			0.124 ^b^			0.109 ^b^
Right-sided hemiparesis	2.27	0.30		2.29	0.55		1.51	0.46		2.21	0.39	
Left-sided hemiparesis	2.26	0.40		2.23	0.61		1.72	0.68		2.33	0.46	
Age		0.08 ^c^	0.436 ^c^		−0.04 ^c^	0.677 ^c^		0.11 ^c^	0.260 ^c^		0.00 ^c^	0.980 ^c^
Number of children		−0.10 ^c^	0.305 ^c^		−0.21 ^c^	0.029 ^c^		0.07 ^c^	0.486 ^c^		−0.08 ^c^	0.396^c^
Family members living in the same house		−0.14 ^c^	0.144 ^c^		−0.17 ^c^	0.076 ^c^		0.03 ^c^	0.787 ^c^		0.00 ^c^	0.976 ^c^
Duration of care (months)		−0.05 ^d^	0.604 ^d^		−0.03 ^d^	0.728 ^d^		−0.03 ^d^	0.775 ^d^		−0.14 ^d^	0.161 ^d^
**Caregivers**												
Sex			0.873 ^a^			0.097 ^a^			0.216 ^a^			0.290 ^a^
Male	2.24	0.41		2.11	0.60		1.70	0.68		2.19	0.39	
Female	2.26	0.32		2.30	0.55		1.56	0.52		2.29	0.45	
Relationship with patient			0.662 ^b^			0.753 ^b^			0.310 ^b^			0.304 ^b^
Spouse	2.23	0.36		2.26	0.65		1.52	0.58		2.23	0.45	
Child	2.26	0.32		2.19	0.44		1.67	0.57		2.22	0.39	
Other	2.32	0.38		2.31	0.55		1.73	0.59		2.41	0.47	
Educational attainment			0.026 ^a^			0.846 ^a^			0.002 ^a^			0.001 ^a^
Uneducated/Primary	2.15	0.29		2.23	0.54		1.37	0.46		2.07	0.32	
Higher/University	2.31	0.37		2.25	0.59		1.73	0.60		2.35	0.46	
Married			0.725 ^a^			0.888 ^a^			0.546 ^a^			0.926 ^a^
No	2.27	0.34		2.26	0.51		1.66	0.55		2.25	0.41	
Yes	2.25	0.35		2.24	0.59		1.58	0.59		2.26	0.45	
Employment			0.296 ^b^			0.782 ^b^			0.093 ^b^			<0.001 ^b^
Working	2.30	0.30		2.26	0.52		1.68	0.53		2.36	0.37	
Retired	2.26	0.37		2.21	0.58		1.64	0.65		2.32	0.44	
Other	2.14	0.35		2.31	0.64		1.33	0.28		1.87	0.34	
Family income (EUR)			0.017 ^a^			0.555 ^a^			0.008 ^a^			<0.001 ^a^
<10,000	2.18	0.33		2.21	0.55		1.47	0.49		2.09	0.38	
≥10,000	2.34	0.35		2.28	0.59		1.76	0.64		2.45	0.42	
Self-perception of health			0.059 ^a^			0.877 ^a^			0.021 ^a^			0.003 ^a^
Bad/Moderate	2.20	0.33		2.25	0.53		1.50	0.52		2.16	0.42	
Good	2.33	0.37		2.23	0.63		1.76	0.64		2.40	0.43	
Age		0.05 ^c^	0.635 ^c^		−0.05 ^c^	0.632 ^c^		0.05 ^c^	0.599 ^c^		0.03 ^c^	0.737 ^c^
Number of children		0.02 ^c^	0.822 ^c^		−0.02 ^c^	0.825 ^c^		−0.03 ^c^	0.795 ^c^		0.04 ^c^	0.673 ^c^
Duration of care (months)		−0.06 ^d^	0.503 ^d^		−0.07 ^d^	0.477 ^d^		−0.03 ^d^	0.782 ^d^		−0.12 ^d^	0.200 ^d^
Daily caring hours		−0.29 ^c^	0.002 ^c^		−0.14 ^c^	0.158 ^c^		−0.16 ^c^	0.097 ^c^		−0.22 ^c^	0.020 ^c^
Caregiving Outcomes Scale (BCOS)		0.25 ^c^	0.008 ^c^		0.15 ^c^	0.117 ^c^		0.14 ^c^	0.14 ^c^		0.17 ^c^	0.08 ^c^
Barthel Index		0.24 ^c^	0.011 ^c^		0.26 ^c^	0.007 ^c^		0.12 ^c^	0.23 ^c^		0.26 ^c^	0.007 ^c^
Center for Epidemiological Studies Depression (CES-D)		−0.11 ^c^	0.234 ^c^		−0.06 ^c^	0.559 ^c^		−0.07 ^c^	0.452 ^c^		−0.03 ^c^	0.776 ^c^
Personal Resource Questionnaire (PRQ 2000)		0.49 ^c^	<0.001 ^c^		0.23 ^c^	0.015 ^c^		0.29 ^c^	0.002 ^c^		0.31 ^c^	0.001 ^c^
	**Spiritual Growth**			**Interpersonal Relations**						**Stress Management**		
**Stroke Survivors**	**Mean**	**SD**	***p*** **-Value**	**Mean**		**SD**		***p*-Value**		**Mean**	**SD**	***p*** **-Value**
Sex			0.630 ^a^					0.188 ^a^				0.056 ^a^
Male	2.63	0.41		2.72		0.43				1.97	0.38	
Female	2.59	0.46		2.60		0.45				2.12	0.40	
Educational attainment			0.594 ^a^					0.884 ^a^				0.568 ^a^
Uneducated/Primary	2.59	0.47		2.66		0.45				2.06	0.40	
Higher/University	2.64	0.37		2.67		0.45				2.02	0.40	
Married			0.677 ^a^					0.557 ^a^				0.180 ^a^
No	2.63	0.50		2.70		0.45				2.11	0.43	
Yes	2.60	0.39		2.64		0.44				2.01	0.38	
Family income (EUR)			0.915 ^a^					0.121 ^a^				0.922 ^a^
<10,000	2.61	0.42		2.60		0.45				2.04	0.40	
≥10,000	2.61	0.45		2.74		0.43				2.05	0.40	
Equipment for disabled			0.121 ^a^					0.340 ^a^				0.243 ^a^
Yes	2.52	0.45		2.61		0.42				1.98	0.36	
No	2.66	0.41		2.69		0.46				2.08	0.41	
Diagnosis			0.133 ^b^					0.217 ^b^				0.518 ^b^
Right-sided hemiparesis	2.70	0.39		2.74		0.46				2.08	0.37	
Left-sided hemiparesis	2.55	0.45		2.60		0.44				2.03	0.43	
Age		0.08 ^c^	0.435 ^c^			0.00 ^c^		0.960 ^c^			0.25 ^c^	0.008 ^c^
Number of children		−0.07 ^c^	0.470 ^c^			−0.08 ^c^		0.410 ^c^			−0.04 ^c^	0.669 ^c^
Family members living in the same house		−0.17 ^c^	0.073 ^c^			−0.15 ^c^		0.116 ^c^			−0.16 ^c^	0.089 ^c^
Duration of care (months)		−0.03 ^d^	0.767 ^d^			0.03 ^d^		0.736 ^d^			0.08 ^d^	0.380 ^d^
**Caregivers**												
Sex			0.673 ^a^					0.275 ^a^				0.033 ^a^
Male	2.63	0.49		2.59		0.50				2.16	0.43	
Female	2.60	0.40		2.69		0.41				1.99	0.37	
Relationship with patient			0.987 ^b^					0.671 ^b^				0.713 ^b^
Spouse	2.62	0.38		2.62		0.43				2.01	0.39	
Child	2.60	0.50		2.69		0.44				2.08	0.42	
Other	2.60	0.41		2.72		0.51				2.05	0.36	
Educational attainment			0.223 ^a^					0.609 ^a^				0.084 ^a^
Uneducated/Primary	2.54	0.41		2.63		0.47				1.95	0.34	
Higher/University	2.65	0.44		2.68		0.44				2.09	0.42	
Married			0.617 ^a^					0.532 ^a^				0.400 ^a^
No	2.57	0.48		2.71		0.49				2.10	0.35	
Yes	2.62	0.42		2.65		0.43				2.03	0.41	
Employment			0.484 ^b^					0.630 ^b^				0.713 ^b^
Working	2.68	0.42		2.72		0.41				2.00	0.40	
Retired	2.58	0.43		2.63		0.45				2.07	0.40	
Other	2.56	0.47		2.64		0.48				2.03	0.39	
Family income (EUR)			0.467 ^a^					0.226 ^a^				0.289 ^a^
<10,000	2.58	0.41		2.61		0.46				2.01	0.36	
≥10,000	2.64	0.46		2.72		0.42				2.09	0.44	
Self-perception of health			0.035 ^a^					0.847 ^a^				0.078 ^a^
Bad/Moderate	2.54	0.43		2.67		0.47				1.99	0.34	
Good	2.71	0.40		2.65		0.41				2.13	0.46	
Age		0.09 ^c^	0.364 ^c^			−0.04 ^c^		0.698 ^c^			0.16 ^c^	0.089 ^c^
Number of children		0.11 ^c^	0.249 ^c^			0.04 ^c^		0.652 ^c^			−0.05 ^c^	0.642 ^c^
Duration of care (months)		−0.10 ^d^	0.297 ^d^			0.06 ^d^		0.532 ^d^			0.08 ^d^	0.438 ^d^
Daily caring hours		−0.22 ^c^	0.019 ^c^			−0.32 ^c^		0.001 ^c^			−0.27 ^c^	0.005 ^c^
Caregiving Outcomes Scale (BCOS)		0.29 ^c^	0.002 ^c^			Outcomes Scale (BCOS)		0.049^c^			Outcomes Scale (BCOS)	0.05 ^c^
Barthel Index (BI)		0.02 ^c^	0.837 ^c^			0.28 ^c^		0.003 ^c^			0.11 ^c^	0.26 ^c^
CES-D		−0.15 ^c^	0.11 ^c^			−0.01 ^c^		0.905 ^c^			−0.22 ^c^	0.02 ^c^
Personal Resource Questionnaire (PRQ 2000)		0.48 ^c^	<0.001 ^c^			0.62 ^c^		<0.001 ^c^			0.26 ^c^	0.007 ^c^

^a^*t*-test; ^b^ analysis of variance; ^c^ Pearson correlation coefficient; ^d^ Spearman correlation coefficient.

**Table 4:** Multivariate analysis of HPLP-II subscales.

Dependent variables	Beta	SE	*p*-Value
Independent variables			
**HPLP- II**			
Secondary/tertiary versus uneducated/primary education		0.06	0.048
Personal Resource Questionnaire (PRQ)	0.01	0.002	0.000
Hours of patient care	−0.01	0.005	0.025
**Health Responsibility**			
Personal Resource Questionnaire (PRQ)	0.010	0.004	0.013
Barthel Index (BI)	0.005	0.002	0.006
**Physical Activity**			
Secondary/tertiary versus uneducated/primary education	0.33	0.11	0.003
Personal Resource Questionnaire (PRQ)	0.011	0.004	0.003
**Nutrition**			
Secondary/tertiary versus uneducated/primary education	0.19	0.08	0.016
Personal Resource Questionnaire (PRQ)	0.008	0.003	0.005
Barthel Index (BI)	0.003	0.001	0.008
Annual income ≥EUR 10,000 versus <EUR 10,000	0.24	0.08	0.002
**Spiritual Growth**			
Good health condition of the caregiver versus poor/moderate	0.15	0.07	0.045
Personal Resource Questionnaire (PRQ)	0.015	0.003	<0.001
**Interpersonal Relations**			
Personal Resource Questionnaire (PRQ)	0.020	0.002	<0.001
Barthel Index (BI)	0.004	0.001	<0.001
**Stress Management**			
Men versus women	0.19	0.07	0.012
Personal Resource Questionnaire (PRQ)	0.007	0.003	0.008
Patient’s age	0.008	0.003	0.003
Hours of patient care	−0.02	0.01	0.007

## Data Availability

The original data presented in this study are available by request to the authors.

## References

[1] Johnson C.O., Nguyen M., Roth G.A., Nichols E., Alam T., Abate D., Abd-Allah F., Abdelalim A., Abraha H.N., Abu-Rmeileh N.M. (2019). Global, regional, and national burden of stroke, 1990–2016: A systematic analysis for the global burden of disease study 2016. Lancet Neurol..

[2] Roth G.A., Feigin V.L., Nguyen G., Cercy K., Johnson C.O., Alam T., Parmar P.G., Abajobir A.A., Abate K.H., Abd-Allah F. (2018). Global, regional, and country-specific lifetime risks of stroke, 1990 and 2016. N. Engl. J. Med..

[3] van Mierlo M., van Heugten C., Post M.W.M., Hoekstra T., Visser-Meily A. (2018). Trajectories of health-related quality of life after stroke: Results from a one-year prospective cohort study. Disabil. Rehabil..

[4] Hayashi Y., Hai H.H., Tai N.A. (2013). Assessment of the needs of caregivers of stroke patients at state-owned acute-care hospitals in southern Vietnam, 2011. Prev. Chronic Dis..

[5] Costa T.F., Costa K.N., Martins K.P., Fernandes M., Brito S. (2015). Burden over family caregivers of elderly people with stroke. Esc. Anna Nery.

[6] Kable A., Pond D., Baker A., Turner A., Levi C. (2018). Evaluation of discharge documentation after hospitalization for stroke patients discharged home in Australia: A cross-sectional, pilot study. Nurs. Health Sci..

[7] Akosile C.O., Okoye E.C., Nwankwo M.J., Akosile C.O., Mbada C.E. (2011). Quality of life and its correlates in caregivers of stroke survivors from a Nigerian population. Qual. Life Res..

[8] Lutz B.J., Young M., Cox K.J., Martz C., Creasy K.R. (2011). The crisis of stroke: Experiences of patients and their family caregivers. Top. Stroke Rehabil..

[9] Pai H.C., Tsai Y.C. (2016). The effect of cognitive appraisal on quality of life of providers of home care for patients with stroke. J. Neurosci. Nurs..

[10] Em S., Bozkurt M., Caglayan M., Cevik F.C., Kaya C., Oktayoglu P., Nas K. (2017). Psychological health of caregivers and association with functional status of stroke patients. Top. Stroke Rehabil..

[11] Rawat M., Sharma R., Goel D. (2017). Burden of stroke survivors on caregiver and quality of life. Int. J. Curr. Res..

[12] Kepic M., Randolph A., Hermann-Turner K.M. (2019). Care for caregivers: Understanding the need for caregiver support. Adultspan J..

[13] Pitthayapong S., Thiangtam W., Powwattana A., Leelacharas S., Waters C.M. (2017). A Community Based Program for Family Caregivers for Post Stroke Survivors in Thailand. Asian Nurs. Res..

[14] Ross A., Sundaramurthi T., Bevans M. (2013). A labor of love: the influence of cancer caregiving on health behaviors. Cancer Nurs..

[15] Tang Y.Y., Chen S.P. (2002). Health promotion behaviors in Chinese family caregivers of patients with stroke. Health Promot. Int..

[16] Clark P.C., Dunbar S.B., Shields C.G., Viswanathan B., Aycock D.M., Wolf S.L. (2004). Influence of stroke survivor characteristics and family conflict surrounding recovery on caregivers’ mental and physical health. Nurs. Res..

[17] Yu Y., Hu J., Efird J.T., Mccoy T.P. (2013). Social support, coping strategies and health-related quality of life among primary caregivers of stroke survivors in China. J. Clin. Nurs..

[18] Caro C.C., Mendes P.V.B., Costa J.D., Nock L.J., da Cruz D.M.C. (2017). Independence and cognition post-stroke and its relationship to burden and quality of life of family caregivers. Top. Stroke Rehabil..

[19] Bailes C.O., Kelley C.M., Parker N.M. (2016). Caregiver burden and perceived health competence when caring for family members diagnosed with Alzheimer’s disease and related dementia. J. Am. Assoc. Nurse Pract..

[20] Bauer J.M., Sousa-Poza A. (2015). Impacts of informal caregiving on caregiver employment, health, and family. JPA.

[21] Bianchi M., Flesch L.D., Alves E.V., Batistoni S.S., Neri A.L. (2016). Zarit Burden Interview Psychometric Indicators Applied in Older People Caregivers of Other Elderly. Rev. Lat. Am. Enfermagem..

[22] Chow S.K., Wong F.K., Poon C.Y. (2007). Coping and caring: support for family caregivers of stroke survivors. J. Clin. Nurs..

[23] TariMoradi A., Ahadi H. (2014). Survey of depression, anxiety and physical health of caregivers to elders with aged and brain stroke. Alborz Univ. Med. J..

[24] Walker S.N., Hill-Polerecky D.M. (1997). Psychometric Evaluation of Health Promoting Lifestyle Profile II.

[25] Bakas T., Champion V. (1999). Development and psychometric testing of the Bakas caregiving outcomes scale. Nurs. Res..

[26] Govina O., Kotronoulas G., Mystakidou K., Giannakopoulou M., Galanos A., Patiraki E. (2013). Validation of the revised Bakas caregiving outcomes scale in Greek caregivers of patients with advanced cancer receiving palliative radiotherapy. Supportive Care Cancer.

[27] Radloff L.S. (1977). The CES-D scale: A self-report depression scale for research in the general population. Appl. Psychol. Meas..

[28] Weinert C., Strickland O., Dilorio C. (2003). Measuring social support: PRQ 2000. Measurement of Nursing Outcomes.

[29] Mahoney F.I., Barthel D.W. (1965). Functional evaluation: The barthel index. MD State Med. J..

[30] Sisk R.J. (2000). Caregiver burden and health promotion. Int. J. Nurs. Stud..

[31] Hulme P.A., Walker S.N., Effle K.J., Jorgensen L., McGowan M.G., Nelson J.D., Pratt E.N. (2003). Health-promoting lifestyle behaviors of spanish-speaking hispanic adults. J. Transcult. Nurs..

[32] Mirghafourvand M., Baheiraei A., Nedjat S., Mohammadi E., Charandabi S.M., Majdzadeh R. (2015). A population-based study of health-promoting behaviors and their predictors in Iranian women of reproductive age. Health Promot. Int..

[33] Jiang S.S., Shen L.P., Ruan H.F., Li L., Gao L.L., Wan L.H. (2014). Family function and health behaviours of stroke survivors. Int. J. Nurs. Sci..

[34] Pinar R., Celik R., Bahcecik N. (2009). Reliability and construct validity of the health-promoting lifestyle profile II in an adult Turkish population. Nurs. Res..

[35] Teng H.L., Yen M., Fetzer S. (2010). Health promotion lifestyle profile-II: Chinese version short form. J. Adv. Nurs..

[36] Mohamadian H., Ghannaee M., Kortdzanganeh J., Meihan L. (2013). Reliability and construct validity of the Iranian version of health-promoting lifestyle profile in a female adolescent population. Int. J. Prev. Med..

[37] Tsai P.C., Yip P.K., Tai J.J., Lou M.F. (2015). Needs of family caregivers of stroke patients: A longitudinal study of caregivers’ perspectives. Patient Prefer. Adherence.

[38] Camak D.J. (2015). Addressing the burden of stroke caregivers: A literature review. J. Clin. Nurs..

[39] Bugge C., Alexander H., Hagen S. (1999). Stroke patients’ informal caregivers: Patient, caregiver, and service factors that affect caregiver strain. Stroke.

[40] McCullagh E., Brigstocke G., Donaldson N., Kalra L. (2005). Determinants of caregiving burden and quality of life in caregivers of stroke patients. Stroke.

[41] Nelson M.M., Smith M.A., Martinson B.C., Kind A., Luepker R.V. (2008). Declining patient functioning and caregiver burden/health: The Minnesota stroke survey-quality of life after stroke study. Gerontologist.

[42] Adams M.H., Bowden A.G., Humphrey D.S., McAdams L.B. (2000). Social support and health promotion lifestyles of rural women. Online J. Rural Nurs. Health Care.

[43] Haya M.A.N., Ichikawa S., Wakabayashi H., Takemura Y. (2018). Family caregivers’ perspectives for the effect of social support on their care burden and quality of life: A mixed-method study in rural and sub-urban central Japan. Tohoku J. Exp. Med..

[44] Melchiorre M.G., Chiatti C., Lamura G., Torres-Gonzales F., Stankunas M., Lindert J., Ioannidi-Kapolou E., Barros H., Macassa G., Soares J.F.J. (2013). Social support, socio-economic status, health and abuse among older people in seven European countries. PLoS ONE.

[45] Govina O., Vlachou E., Kalemikerakis I., Papageorgiou D., Kavga A., Konstantinidis T. (2019). Factors associated with anxiety and depression among family caregivers of patients undergoing palliative radiotherapy. Asia-Pac. J. Oncol. Nurs..

[46] International Family Nursing Association (INFA) (2018). Position Statement on Graduate Family Nursing Education.

[47] Reeves K.W., Bacon K., Fredman L. (2012). Caregiving associated with selected cancer risk behaviors and screening utilization among women: Cross-sectional results of the 2009 BRFSS. BMC Public Health.

[48] Son K.Y., Park S.M., Lee C.H., Choi G.J., Lee D., Jo S., Lee S.H., Cho B. (2011). Behavioral risk factors and use of preventive screening services among spousal caregivers of cancer patients. Supportive Care Cancer.

[49] Geyer S., Hemström Ö., Peter R., Vågerö D. (2006). Education, income, and occupational class cannot be used interchangeably in social epidemiology. Empirical evidence against a common practice. J. Epidemiol. Community Health.

[50] Beesley V.L., Price M.A., Webb P.M. (2011). Loss of lifestyle: Health behaviour and weight changes after becoming a caregiver of a family member diagnosed with ovarian cancer. Supportive Care Cancer.

[51] Nocon M., Keil T., Willich S.N. (2007). Education, income, occupational status and health risk behaviour. J. Public Health.

[52] Gonzalez C., Bakas T. (2013). Factors associated with stroke survivor behaviors as identified by family caregivers. Rehabil. Nurs..

[53] Efi P., Fani K., Eleni T., Stylianos K., Vassilios K., Konstantinos B., Chrysoula L., Kyriaki M. (2017). Quality of life and psychological distress of caregivers’ of stroke people. Acta Neurol. Taiwan.

